# Comparing the Relative Strengths of EEG and Low-Cost Physiological Devices in Modeling Attention Allocation in Semiautonomous Vehicles

**DOI:** 10.3389/fnhum.2019.00109

**Published:** 2019-03-29

**Authors:** Dean Cisler, Pamela M. Greenwood, Daniel M. Roberts, Ryan McKendrick, Carryl L. Baldwin

**Affiliations:** ^1^Department of Psychology, George Mason University, Fairfax, VA, United States; ^2^Northrop Grumman, Falls Church, VA, United States

**Keywords:** low-cost technology, attention, alpha-band, semiautonomous vehicles, eye-tracking, electrocardiography

## Abstract

As semiautonomous driving systems are becoming prevalent in late model vehicles, it is important to understand how such systems affect driver attention. This study investigated whether measures from low-cost devices monitoring peripheral physiological state were comparable to standard EEG in predicting lapses in attention to system failures. Twenty-five participants were equipped with a low-fidelity eye-tracker and heart rate monitor and with a high-fidelity NuAmps 32-channel quick-gel EEG system and asked to detect the presence of potential system failure while engaged in a fully autonomous lane changing driving task. To encourage participant attention to the road and to assess engagement in the lane changing task, participants were required to: (a) answer questions about that task; and (b) keep a running count of the type and number of billboards presented throughout the driving task. Linear mixed effects analyses were conducted to model the latency of responses reaction time (RT) to automation signals using the physiological metrics and time period. Alpha-band activity at the midline parietal region in conjunction with heart rate variability (HRV) was important in modeling RT over time. Results suggest that current low-fidelity technologies are not sensitive enough by themselves to reliably model RT to critical signals. However, that HRV interacted with EEG to significantly model RT points to the importance of further developing heart rate metrics for use in environments where it is not practical to use EEG.

## Introduction

Semiautonomous driving systems or “partial driving automation” (SAE Level 2; SAE International, 2016) are driver assistance systems that are increasingly available in passenger vehicles, with conditional driving automation (SAE level 3) still largely under development. As recently pointed out by Eriksson and Stanton (2017), SAE level 2 is commonly confused with highly automated driving, when in fact the semiautonomous level requires drivers to monitor the automation. For both SAE levels 2 and 3, drivers must be prepared to intervene when system limitations and failures occur. These systems are intended to be advanced driver assistance systems (ADASs) and thus are not intended to supplant the need for drivers to maintain vigilant attention and intervene when necessary.

ADAS in passenger vehicles are urgently needed. Highway fatalities in the US declined steadily for five decades but increased more than 10% in the first 6 months of 2016 with only a slight decline (0.8%) from that peak in 2017 (NHTSA’s National Center for Statistics and Analysis, 2017). Overall, the 2016 and 2017 fatality numbers are a troubling reversal of decades of improvement in highway fatalities. Importantly, an estimated 94% of fatal crashes are attributable to driver error, with 41% of those errors being recognition errors including inattention, internal and external distractions, and inadequate surveillance (Singh, 2015). The advent of semiautonomous systems in vehicles is already reducing crashes by reducing driver error. Automatic emergency braking reduced rear-end crashes by about 40% (Cicchino, 2017) and rear cross-traffic alerts reduced backing crashes by about 32% (Cicchino, 2018).

Despite the potential benefit for automation to reduce vehicle crashes, automation can have unpredictable effects on drivers. Increased vehicle automation changes how drivers pay attention and tend to decrease situation awareness (Sarter et al., 1997; Endsley, 2017). People use automation when they should not, over-rely on automation, over-trust automation, and fail to monitor automation closely (Parasuraman and Riley, 1997). In a prior meta-analysis, a greater degree of automation was found to be associated with reduced ability to recover from a system failure (Onnasch et al., 2014). Importantly, increased levels of vehicle automation shift the driver’s role from one of active control to one of a supervisor of the automation (van den Beukel et al., 2016). It is imperative to understand how advanced vehicle automation affects the safety of drivers and passengers.

Although ADASs do reduce crashes, they also have a number of known operational limits. Misunderstanding or over-trust in these systems may result in drivers failing to monitor the automation and subsequently failing to detect critical signals related to the system’s functionality (Parasuraman and Manzey, 2010). There have been recent news reports of fatal Tesla crashes that occurred when the automation failed to detect obstacles during a period when the driver was not monitoring the automation (CNBC, 2018). Current ADASs are not designed to brake effectively during “cut-in,” “cut-out,” or crossing-path scenarios. Pedestrian detection systems do not detect all pedestrians, notably those carrying large packages. These limits render driver inattention hazardous in all partially automated SAE 2 vehicles. Now that most new vehicles are equipped with some automation, it is important to understand how drivers respond to signals indicating automation disengagement. Inattentive drivers may require more urgent warnings—warnings that could annoy or startle the attentive driver. Therefore, warnings of automation faltering or failing should be tailored to the driver’s attentional state to be most effective. Further, there is increasing recognition that under some conditions, safety considerations may require automation to shut itself off to protect an inattentive driver. Such systems would depend on non-invasive sensors able to reliably detect driver attentional state. A major focus of the current work is to understand the predictive capabilities of non-invasive low-cost sensors, compared to well established but expensive and relatively cumbersome methods such as multi-channel EEG.

EEG obtained with standard EEG recording equipment has been shown to be sensitive to attentional state and is often considered the defacto physiological measure for attention. Previous EEG studies using high-fidelity EEG systems, have reported that alpha-band activity increases just before errors in processing that stimuli (Mazaheri et al., 2009; O’Connell et al., 2009; Brouwer et al., 2012; Ahn et al., 2016; Aghajani et al., 2017; Zhang et al., 2017). Increased prestimulus alpha-band has also been associated with mind wandering during driving (Baldwin et al., 2017). Although EEG is well-established as a measure of attention, it may not be practical for use in vehicles insofar as real-time scalp recording and analysis of alpha-band power would be needed. Portable EEG systems have shown promise in their ability to monitor driver engagement and drowsiness in a simulator study (Johnson et al., 2011). Even though portable EEG systems may be capable in field settings, they are expensive compared to other portable physiological measuring systems, thereby adding to consumer costs. Lower-cost technology systems exist for monitoring driver state that are more robust and less cumbersome than EEG and thus more likely to be adapted and installed into vehicles. For example, the General Motors Cadillac 2018 and 2019 CT6 models offer a super cruise feature that includes an infrared eye-tracking system which is used by the automation to determine driver attention (Clerkin, 2017). Similarly, low-cost, reliable heart rate monitors with signal quality comparable to that produced by Zyphr^TM^ and KardiaMobile, could potentially be integrated into vehicles to record drivers’ heart electrical activity (ECG). This raises the question of whether sufficient classification sensitivity to the attentional state can be achieved with low-fidelity, low-cost sensors such as heart-rate monitors and eye-trackers?

An existing body of research has investigated the use of metrics other than EEG to monitor operator state. For example, metrics of cardiovascular activity have been used to assess constructs such as mental workload, fatigue, and operator stress. In general, both heart rate increases and heart rate variability (HRV) decreases have been associated with increased mental effort (Mulder, 1992; Wilson, 1992). For example, Stuiver et al. (2014) found that 40 s periods of HRV were sensitive to increased effort expenditure due to driving in fog vs. clear visibility, with fog-inducing decreased HRV. Mehler et al. (2012) found that heart rate and skin conductance level increase as cognitive demand increases. HRV has also been used to classify fatigue during simulated driving (Patel et al., 2011). Metrics of HRV have been found to index changes in mental effort over time as participants adapt to a task and change task strategies and performance criteria. Short periods of high HRV reflecting primarily parasympathetic influences may, therefore, serve as a sensitive index of fluctuations in task effort and temporarily lowered levels of effort on a trial by trial basis (Thayer et al., 2012). HRV as a workload measure is generally most sensitive in the mid-range, particularly around 0.10 Hz area (Mulder, 1992). The mid-range is most sensitive to the amount of mental effort invested in the task, not task complexity, *per se*. Hogervorst et al. (2014) directly compared three measures of HRV used to index workload: (a) high-frequency HRV measured in root mean square of successive differences (RMSSDs); (b) the spectral power in the range 0.15–0.5 Hz of the ECG R to R intervals; and (c) mid-frequency variability with spectral power between 0.07 and 0.15 Hz of the ECG R to R intervals. It should be noted that the third measure would be categorized as low frequency according to the (Task Force of the European Society of Cardiology the North American Society of Pacing Electrophysiology, 1996). Hogervorst et al. (2014), found that, apart from EEG, only respiration frequency and RMSSD produced a significant classification of workload.

Metrics of eye movements have also shown promise in recent years as indices of attention. Metrics obtained from eye trackers, such as fixations, horizontal spread of fixations, and gaze concentration have been used successfully to index attention in several recent driving investigations. For example, Wang et al. (2014) compared a number of different eye gaze metrics and found that horizontal gaze concentration derived from the standard deviation of horizontal gaze position was robust and sensitive to changes to cognitive demand during driving on actual roads. Research by Fridman et al. (2018) used in-vehicle video recordings of eye movements in conjunction with either, Hidden Markov Models or three-dimensional convolutional neural network, to classify driver cognitive load during driving on an actual highway. Likewise, in a simulated vehicle automation task, Louw and Merat (2017) found horizontal gaze dispersion to be sensitive to increased task demand stemming from secondary task engagement. Dehais et al. (2011) and Zeeb et al. (2015) found that gaze concentration was a sensitive index of attentional focusing, found to predict the speed of “take-over” from automation.

Combinations of physiological measures have shown particular promise. For example, combinations of EEG, eye-tracking, and HRV have been used to: (a) classify operator states (Hogervorst et al., 2014); (b) determine whether a driver is on-task or mind wandering (Baldwin et al., 2017); and (c) to successfully adapt automation to improve driver performance (Wilson and Russell, 2003a,b). Hogervorst et al. (2014) provided a partial comparison, reporting that EEG measures obtained the highest classification accuracy compared to eye, heart, and respiratory measures. When EEG was combined with eye measures (pupil size and eyeblinks) there was not a significant improvement over EEG alone as predictors of workload in an n-back working memory task.

In light of evidence that RMSSD (Hogervorst et al., 2014) and eye-gaze (Dehais et al., 2011; Wang et al., 2014) were both found to be effective in predicting driver attentiveness, we hypothesized that these two measures in combination and when obtained from low-cost equipment could be as sensitive in predicting driver performance in a simulator during automated driving as EEG alpha-band, obtained from high-fidelity EEG equipment.

## Materials and Methods

### Participants

Twenty-five participants were recruited through the George Mason University undergraduate research pool, in exchange for course credit. Participant requirements were to be above 18 years of age, have normal or corrected to normal vision and hearing, not currently taking psychoactive medications, and have a valid United States driver’s license. Participants were also asked to not wear heavy eye makeup the day of their scheduled appointment or wear braids, wigs, or hair extensions as they affect contact between EEG electrodes and the scalp. In order to increase enrollment in the study, in addition to course credit, some participants were given a $15.00 bonus upon completion of their scheduled session. Table 1 provides an overview of participant demographic information.

**Table 1:** Participant demographics.

Total participants	25	Female (12)
Age	Range: 18–39	Mean: 22.6 (6.01 SD)
Driving experience (in months)	Range: 6–270	Mean: 57.13 (60.75 SD)

### Materials

#### Simulated Drives

Five fully autonomous drives were programmed using a low-fidelity desktop simulator containing Internet Screen Assembler pro version 20 and Real Time Technologies Sim Creator version 3.2 simulator software on a Windows 7 computer with 64-bit operating system. Each of the drives was displayed on a Dell Monitor with screen size measuring 52 cm in length and 32.5 cm in height with a screen resolution of 1,920 × 1,200 pixels. Each of the drives was programmed to complete an automated lane changing task, adapted from Mattes (2003) and lasted approximately 10 min in duration. The 10-min duration was due to limitations in the Sim Creator software. During the drives, participants were instructed to respond with serial button presses every time the system indicated there was an automation failure. System functionality was represented by right or left facing arrows, appearing in the bottom right corner of the monitor that varied in the gradient of the color red to green and appeared on average every 13 s, with a jitter ±2 s resulting in 4–5 lane changes per minute. Arrow duration was 150 ms. System reliability was indicated by the amount of red at the tip of the arrow. Arrows representing reliable system functionality, Reliable Automation Arrows (presented on 80% of trials) indicated the system was operating normally (the base of the arrow was green with a small amount of red at the tip). After the presentation of a Reliable Automation Arrow, the vehicle would respond by changing lanes correctly. Arrows indicating unreliable system functionality, Unreliable Automation Arrows (presented on 20% of trials or on 10 trials per drive) indicated that the system had failed (the arrow tip was completely filled in with red). After the presentation of the Unreliable Automation Arrow, the vehicle would respond by making one of three possible lane changes. Of the ten Unreliable Automation Arrows, on six of them the vehicle would fail to make a lane change, for two of them the vehicle would respond by making an incorrect lane change (opposite of where the Unreliable Automation Arrow was pointing), and for two the vehicle would make a correct lane change. Participants were told to respond with a button press if the arrow was an Unreliable Automation Arrow then make a second button press to indicate which type of lane change the vehicle made after the presentation of the Unreliable Automation Arrow. Participants were exposed to a total of 50 arrows per 10-min drive.

Two secondary tasks were administered to participants in addition to the lane changing task. The point of these tasks was to keep participants engaged in the driving task and discourage participants from focusing their eyes on the icons in the interface. During each of the time periods, participants were asked to: (a) keep a running count of the number of Coca-Cola and Northrop Grumman signs they encountered; and (b) answer “driver engagement” questions regarding the vehicle’s status such as: speed changes, current lane position, or lane changes. In each time period, there were 25 total billboards and three “driver engagement” questions.

#### Questionnaires

Participants were administered a demographics questionnaire, the Trust Between People and Automation (Jian et al., 2000), Merritt (2011) Trust Scale Items, the Merritt (2011) scale based on Liking Items, and the Propensity to Trust Scale Items (Merritt et al., 2013).

#### EEG Recording

Each participant was equipped with a 40-channel NuAmps EEG cap with silver/silver-chloride electrodes. Data were recorded from a subset of electrodes: Fz, Cz, Pz, Oz, F1, F2, P1, P2, Ground (at location AFz), A1 (the left mastoid, serving as the online reference), and A2 (the right mastoid), as well as EOG electrodes placed above and below the left eye as well as at the outer canthus of both eyes. Data were collected at a sampling rate of 500 Hz with an online high-pass filter of 0.1 Hz and an online low-pass filter of 70 Hz.

#### Eye-Tracking

Gaze dispersion was recorded using the Pupil Pro headset developed by Pupil Labs. This is a low-cost eye-tracker that monitors the participant’s right pupil with a camera as well as the environment with a head-mounted camera. The data was recorded using Pupil Lab recording software. Sensor settings for the cameras were as follow: the pupil camera was set to 640 × 480 with a frame rate of 120 fps maximum resolution and the world camera was set 1,920 × 1,080 with a frame rate of 30 fps maximum resolution.

#### Heart Rate Monitor

A low-cost Zephyr BioPatch heart rate monitor was attached to the participant using ECG electrodes in order to collect heart rate activity during each of the time periods.

#### Lab-Streaming Layer

The lab-streaming layer (LSL) software library^1^ was used to synchronize the timestamps through a network connection between the driving simulation, as well as our physiological devices: the eye-tracker and heart rate monitor.

### Procedure

After providing written informed consent of a protocol approved by George Mason University’s Human Subjects Institutional Review Board, participants were introduced to the heart rate monitor, eye-tracker, the EEG cap. Procedures were used to lower impedance of the scalp EEG electrodes.

#### ECG Setup

Participants were handed the Zephyr Heart Rate Monitor and asked to place it so that it was centered with their sternum so the ECG electrodes could acquire heart rate activity at the fourth intercostal space located at the left and right sternal border. Next, the heart rate monitor was synced with the BioHarness software on a nearby laptop computer.

#### EEG Setup

Next, participants were fitted with the Neuroscan 40 channel EEG cap. Impedance was lowered to 5 kΩ or below by applying electroconductivity gel between the electrodes and the scalp then lightly abrading the scalp using a blunt needle (Luck, 2005). Next participants were shown how excessive movement can introduce noise into EEG waveforms and asked to remain as still in their chair as possible for the duration of the experiment.

#### Eye-Tracking

We used the Pupil Pro headset to monitor eye movements and gaze patterns for the duration of the drives. After placing the headset on each participant, the pupil camera was adjusted to better capture their pupil. Once the camera was able to accurately track the participant’s pupil, they underwent a calibration process in order to synchronize the pupil tracking camera with the world facing camera *via* Pupil Pro software. This allowed us to track the location of the display in order to convert the gaze position to display coordinates. A confidence value is estimated for each sample of eye data that ranges from 0 to 1 indicating a level of certainty that the pupil was accurately identified for that sample. Only samples with confidence at or above 0.8 were used for further data analyses.

After setting up the participant with the physiological metrics, participants were seated 75 cm away from the monitor. At the start of the training drive, each participant was read the instructions aloud and introduced to the controls on the gear shift. Participants were instructed to immediately press the button labeled as *U* as soon as they saw an Unreliable Automation Arrow, then make a second button press indicating the type of error that occurred (*N* = No lane change, *I* = Incorrect lane change, and *C* = Correct lane change). Participants were asked to only respond to the Unreliable Automation Arrows. Participants were also instructed to pay attention to the images on each of the billboards and count the number of times they saw logos for Coca-Cola and Northrop Grumman as well as answer the “Yes” or “No” driver engagement questions (DEQ; e.g., “Speed increased after last arrow?,” “I am currently traveling 67 mph?,” “I am currently in the far right lane?” presented during each trial. Participants were allowed to complete the practice as many times as they needed to feel comfortable responding to the task. After training, participants were then administered the five time periods in counterbalanced order. After each time period participants were asked to report the total number of Coca-Cola and Northrop Grumman billboards to the experimenter. Upon completion of the simulated driving session, participants were administered the questionnaires.

### Data Analysis

#### Heart Rate Variability (HRV)

Data collected from the driving simulator was synced in time with the data collected from the Zephyr Heart Rate Monitor. We sampled the heart rate data starting 10 s before the onset of the Unreliable Automation Arrow until the presentation of the arrow. As reported in Klinger (1978), shifts in thought patterns can happen on average every 14 s. A maximum window of 10 s was chosen for ECG activity as that would allow us to maximize the number of sampled beats per second without extending too far back to potentially sample HRV due to the previous Automation Arrow. Based on previous work by Hogervorst et al. (2014), HRV was the measure of interest because it has been shown to be a robust classifier in identifying low vs. high workload compared to spectrally defined medium and high HRV. We calculated the ECG R-wave peak to peak interval for each trial using the MATLAB wavelet toolbox, using the maximum overlap discrete wavelet transform (MODWT). The squared absolute value of the signal approximation was calculated allowing for the use of an algorithm to identify R peaks for further analysis. Mean R to R was calculated by averaging the time between R peaks (meanRR). HRV was calculated using the RMSSDs.

#### EEG Processing

EEG spectral data were processed using MATLAB with EEGLAB toolbox version 12.0.2.4b (Delorme and Makeig, 2004). EEG channels were mapped using the BESA file, a four shell DIPFIT spherical model of the channel locations. Data were re-referenced to the average of the two mastoid electrodes. Unreliable Automation Arrows were labeled within the waveform of the EEG data. Data were filtered at a high-pass filter of 1 Hz cutoff and 2 Hz transition bandwidth, and a low-pass filter of 40 Hz and 10 Hz transition bandwidth. Data was decomposed *via* independent component analysis (ICA), and components representing blinks or eye movements were visually identified and removed. Electrodes exceeding ±2 standard deviations were identified as artifactual and rejected. Additionally, data exceeding ±100 μV was rejected from the data to remove artifacts caused by large movements or other noise. Data from electrodes rejected due to artifacts that exceeded two standard deviations were subjected to spherical interpolation. Dummy markers were placed in the EEG data 1 s before each unreliable signal event to the presentation of the arrow and the data were epoched to those markers. The 1-s window was chosen to capture the mental state of participants immediately prior to the onset of the Unreliable Automation Arrow. Previous research on what is termed “prestimulus alpha” have shown increases in alpha spectral power, prior to a failure in detecting a signal, using a time window of 800 ms to 1,000 ms prior to stimulus onset (Busch et al., 2009; Mazaheri et al., 2009). Each epoch was linearly detrended, and a hamming windowed Fourier transform was used to convert the data from the time-domain to the frequency domain, as implemented in the MATLAB function pwelch. The data were then converted into decibel power using 10*log10 (power) in order to get a better approximation of the normal distribution. The FFT bin nearest to 10 Hz, here 9.76 Hz, was used to analyze alpha activity at electrodes Pz, Cz, and Fz.

#### Eye-Tracking

Gaze dispersion data collected from the eye-tracker was synced in time with each drive through LSL and sampled 3 s before each onset of an Unreliable Automation Arrow to the presentation of the arrow. This time window was selected in order to maximize the number of sampled eye movements prior to the onset of the Unreliable Automation Arrow while avoiding potential contamination from eye movements that occurred due to the billboard task. Horizontal and vertical gaze dispersion were calculated by computing the standard deviation of a measure of pixels over which the eyes moved for the X (horizontal) or Y (vertical) dimension of the raw data identified with a confidence value of 0.8 or higher. Horizontal and vertical gaze dispersion was then transformed using the natural log of their values (lnX and lnY, respectively) to approximate the normal distribution.

#### Behavioral Data

Responses to the presentation of the Unreliable Automation Arrows and responses indicating the type of error (second button presses) were extracted to assess changes in performance over a time period during the experimental session. Participants were instructed to immediately respond as soon as they saw an Unreliable Automation Arrow. Due to high accuracy shown by participants in identifying Unreliable Automation Arrows, the latency of response to Unreliable Automation Arrows was the measure of interest. We first calculated the grand mean for our entire data set and standard deviation. We set all response times higher than 2,600 ms to equal 2,600 ms. In order to get a better idea of how well participants were able to distinguish between critical events and reliable events, the *A* measure of sensitivity was used. Since the measure of d’ is calculated by taking the difference of hits and false alarms that have been converted from probabilities into z-scores, the inclusion of a 1 or a 0 can lead to a value that does not fall below the ROC curve. Use of non-parametric sensitivity calculated using the *A* statistic, as described in Zhang and Mueller (2005), eliminates the reliance of converting probabilities to z-scores and obtains the measure of sensitivity by calculating the average of the minimum-area and maximum-area proper ROC curves as constrained by false alarms and hits. Analysis of the accuracy of the second button press that indicated the type of lane change the vehicle made (incorrect, correct, or no lane change) were calculated for further analysis in SPSS.

For the billboard task, the probability of hits and false alarms was calculated for each 10 min time period of the drive. The *A* statistic was calculated for further analysis in SPSS. For the DEQs, accuracy was calculated by averaging the responses of the questions for each 10 min episode of the drive. With the trust questionnaire data, statements identified as being negative were reverse coded allowing us to average the scores for further analyses.

## Results

Data were analyzed using SPSS and the R statistical package (R Core Team, 2017). To assess how well participants were able to discriminate between Unreliable Automation and Reliable Automation Arrows, we calculated the *A* statistic for each time period. To assess speed-accuracy tradeoff, a correlation analysis was conducted comparing *A* to reaction time (RT) for the discrimination task. That analysis produced a significant, 2-tailed, negative correlation (*R*^2^ = −0.50, *p* < 0.05), indicating that participants did not slow their responses in order to achieve higher accuracy scores. Since accuracy was at the ceiling for participants, discrimination RT was the behavioral measure of interest.

In order to model RT to Unreliable Automation Arrow over the five time periods, linear-mixed effects models were carried out. These models were constructed using the R package *lme4* (Bates et al., 2012). We conducted interactive models of RT to Unreliable Automation Arrow across the five time periods for each measure (alpha-band × time period, HRV × time period, meanRR × time period, lnX × time period). These were random intercept and slope models. Participant and trial (10 trials in each time period) were random factors. For each variable, only time period significantly modeled RT (*p* < 0.05). Only alpha-band interacted with the time period in modeling RT. A likelihood ratio test (LRT) comparing the interactive model (alpha-band × time period) to a null additive model (alpha-band + time period) produced a significant Chi-square ($X_{\left(1\right)}^{2}$ = 5.251, *p* = 0.0219), suggesting that the interaction was important in modeling RT.

**Figure 1:**
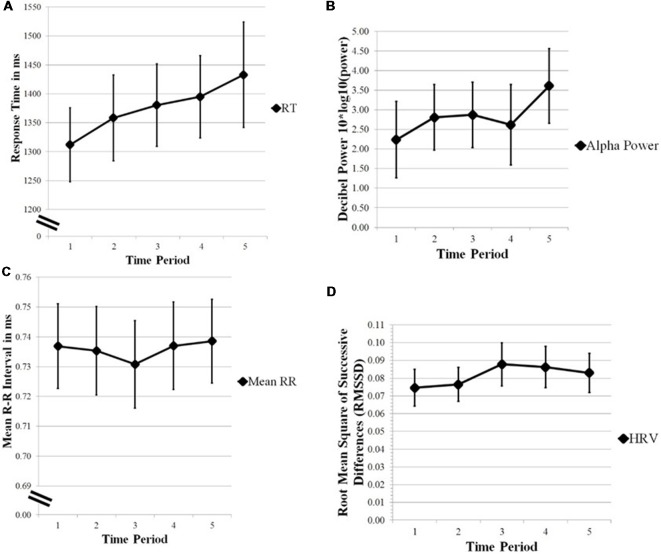
**(A)** Reaction time (RT). **(B)** Alpha-band power. **(C)** Mean RR. **(D)** Heart rate variability (HRV) plotted over 10 min time periods. Error bars are standard error of the mean. Alpha-band power, Mean RR, and HRV are important factors in modeling RT over time.

**Figure 2:**
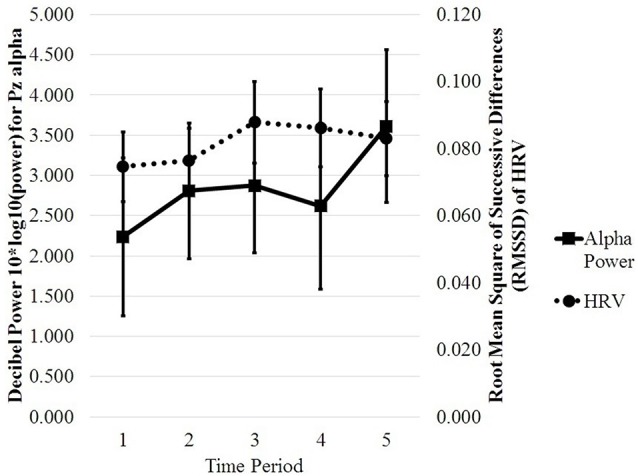
Plotted interaction of Pz Alpha and HRV across time period. Error bars are standard error of the mean. Alpha power increases and HRV decreases at Time Period 5.

Linear-mixed effects models were also used to model RT. An interactive model of RT was constructed with alpha-band, meanRR, HRV, lnX, and time period as fixed factors (Formula: RT ~ 1 + (Pz Alpha + meanRR + HRV + lnX + TimePeriod)^3^ + (1|Participant) + (1|Trial)). Participant and trial (10) were random factors. That model produced two significant interactions (AIC = 2727.7, BIC = 2873.3, *p* < 0.05), indicating the likelihood of alpha-band × time period (*β* = 0.04158) and alpha-band × HRV (*β* = −0.1588) in modeling RT. As horizontal gaze dispersion (lnX) did not contribute significantly to the model, lnX was dropped from the model and a reduced model was fitted (Bolker et al., 2009). The reduced LME was conducted to model RT using alpha-band, meanRR, HRV, and time period as the fixed factors (Formula: RT ~1 + (Pz Alpha + meanRR + HRV + TimePeriod)^3^ + (1|Participant) + (1|Trial)). Interactions were limited to two- and three-way. That model produced a significant three-way interaction (AIC = 2713.2, BIC = 2803.5, *p* < 0.05, marginal *R*^2^ = 0.02, conditional *R*^2^ = 0.42) indicating the likelihood of alpha-band, HRV, and time period (*β* = 0.03861) in modeling RT. The *R*^2^ values, calculated and reported as described in Nakagawa et al. (2017), indicate that 2% of the variance was explained by the fixed factors alone while 42% of the variance was explained by random effects included in the model. The model also produced a significant two-way interaction of MeanRR × Time Period (*β* = −0.03588, *p* < 0.05). A LRT comparing the interactive model (alpha-band × meanRR × HRV × time period) with an additive null model produced a significant Chi-square ($X_{\left(10\right)}^{2}$ = 21.092, *p* = 0.021), indicating the interactions were important in modeling RT. Since the only significant three-way interaction involved alpha-band, HRV, and time period, LRTs were conducted to test the interactions: (a) alpha-band × HRV; (b) alpha-band × Time Period; and (c) HRV × Time Period. The three LRT tests showed that alpha-band × HRV ($X_{\left(9\right)}^{2}$ = 18.649, *p* = 0.0284) and HRV × Time Period ($X_{\left(9\right)}^{2}$ = 19.228, *p* = 0.023) were significant. The interaction of Alpha-band × Time Period was not significant ($X_{\left(9\right)}^{2}$ = 15.809, *p* = 0.071). Considered together, these results indicate that alpha-band, HRV, and time period are important factors in modeling RT, with meanRR a weaker factor. Figure 1 shows the changes for the physiological measures over each time period. Figure 2 provides a visual comparison of alpha-band power and HRV over time period.

### Response to Lane Change Accuracy

Accuracy scores calculated from the second button presses which identified the type of lane change made by the vehicle were submitted to a repeated measures ANOVA to assess the change in accuracy over time. There was no statistical significance in the analysis of changes over time in accuracy of deciding which type of lane change was made by the vehicle (*F*_(4,92)_ = 0.404, *p* = 0.806).

### Billboard Task

**Figure 3:**
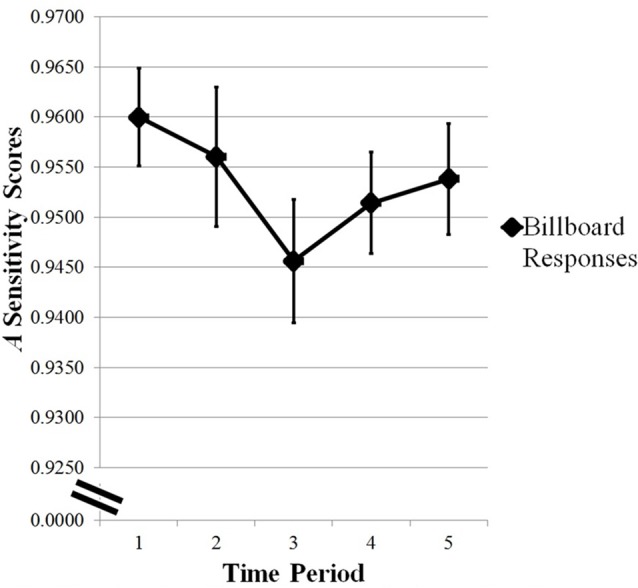
Changes in *A* sensitivity scores for the Billboard task across time period. Error bars are standard error of the mean. Statistical significance was not observed for *A* sensitivity scores across time period.

Preliminary analyses of *A* sensitivity scores were calculated looking at the changes in sensitivity to identifying the Northrup Grumman and Coca Cola billboards over time. The *A* statistic was calculated for the billboard task, as shown in Figure 3. Due to high accuracy for the billboard responses and in the absence of a hypothesis on an effect of the two billboard types, *A* scores were collapsed across Northrup Grumman and Coca Cola billboards. A repeated measures ANOVA was conducted in SPSS looking at changes in *A* sensitivity scores as a function of time. Statistical significance was not observed (*F*_(4,92)_ = 1.495, *p* = 0.210).

### Driver Engagement Questions

**Figure 4:**
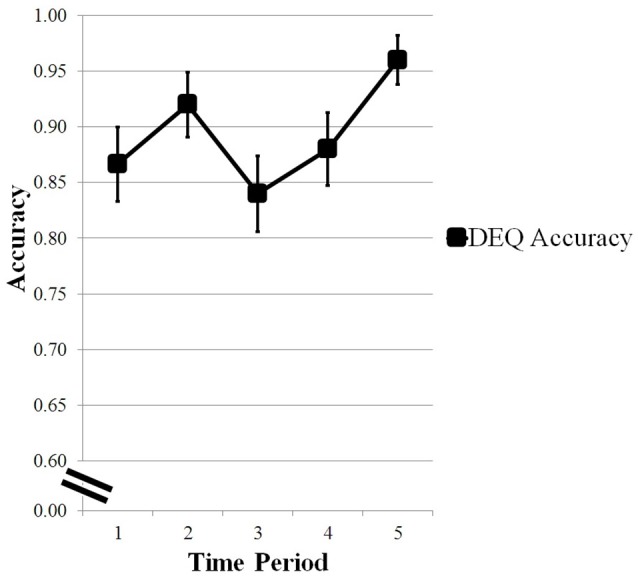
Changes in accuracy scores of Driver Engagement Questions (DEQ) across time period. Error bars are standard error of the means. Marginal effect of time on response accuracy.

Accuracy was calculated for each time period by averaging the responses for the DEQs. As shown in Figure 4, participants increased in accuracy in their responses to the questions before showing a performance decrease at the third time period and an increase in performance for the fourth and fifth time period. A repeated measures ANOVA analyzed the change in accuracy over time. Mauchly’s test of sphericity indicated that the assumption of sphericity had not been violated and therefore sphericity was assumed. There was a marginal effect of time on accuracy of response (*F*_(4,96)_ = 2.353, *p* = 0.059).

### Trust Questionnaires

Five correlation analyses were conducted to assess the relationship between our questionnaires (Trust Between People and Automation, Merritt Trust Scale Items, Merritt scale based on Liking Items, Propensity to Trust Scale Items), physiological metrics selected based on the LME (alpha-band, Mean RR, and HRV), and behavioral metrics (RT and *A*). Of our five correlation analyses, there was a statistically significant negative bivariate correlation between alpha-band activity at midline parietal site Pz and the Merritt et al.’s (2013) Propensity to Trust Scale Items (*r* = −0.430, *p* < 0.05).

## Discussion

We obtained partial support for our hypothesis. We found that HRV interacted with alpha-band activity and time period to model the speed of processing signals of automation unreliability. Gaze dispersion did not model the speed of processing signals of automation unreliability, either alone or in combination with other measures. Mean RR (heart rate measured in R-R intervals) did model RT in interaction with time period but not in interaction with alpha-band or HRV. Our findings confirm previous evidence that prestimulus alpha-band activity is the most effective measure of mental processing (Hogervorst et al., 2014) but extend that work in showing HRV increased the predictive capability of parietal alpha-band. The readiness of the brain to process signals of system unreliability was affected by the combined effects of HRV and alpha-band activity. This evidence that HRV modulates alpha-band activity with consequences for automation signal processing argues for the importance of developing heart rate metrics in operational environments where EEG is not practical.

Regarding the time course, HRV initially increased over the session of autonomous vehicle driving, but then decreased near the end. Based on the existing HRV literature, the effect of workload on HRV depends in part on the duration of the workload demand. Mulder (1992) has argued that the cardiac response to 5–10 min periods of increased workload reflects preparation for fight-or-flight activation of the sympathetic nervous system with increased HR and decreased HRV. In contrast, a short-lasting increase in workload (25–30 s) was reflected in short-lasting increases in heart rate and blood pressure in combination with corresponding decreases in HRV and blood pressure variability (Stuiver et al., 2012). For our task, the workload may have increased when Unreliable Automation Arrow signals were presented. However, the present study measured HRV prior to those unpredictable signals indicating unreliable automation. Therefore we could not determine whether those signals transiently increased workload. The slowing of RT linearly over the session and the initial increase in HRV during autonomous vehicle operation are consistent with an interpretation that workload increased over the session.

HRV has previously been associated with emotional regulation (Appelhans and Luecken, 2008). HRV has been found to be higher in those people who were better able to regulate their emotions in social interactions (Butler et al., 2006) and in marital interactions (Smith et al., 2011). Our finding that high-frequency HRV interacted with alpha-band to model the speed of responding to unreliable signals points to a role for individual differences in emotional response regulation in processing automation signals. Further, in operational environments, it might be interesting to determine whether very low and low-frequency HRV also predicts RT of responding to signals of automation reliability.

RT to the signals of unreliable automation slowed fairly linearly over the 55-min drive. Use of RT to measure processing of signals from automation during a simulated drive is very relevant to the topic of real-world driving of vehicles equipped with ADASs. In ADAS-equipped vehicles in the real world, the driver receives frequent signals from various automation systems [e.g., drowsy driving, lane departure, lane keeping, and (more rarely) sensor failure warnings]. The slowing of RT to automation signals over the simulated driving session could suggest a vigilance decrement. However, the sensitivity index *A* from the discrimination task did not change over the driving session and accuracy of responses to the lane changing task was high. Moreover, the driving session was interrupted briefly every 10 min or so (due to limitations of the software), which would not be conducive to the development of a vigilance decrement. Therefore, we do not interpret our findings of slowed RT as consistent with a vigilance decrement. Workload is another possible explanation for slowing RT to signals of automation. The decrease in accuracy on the DEQs between the second and third time points, despite the high accuracy of the secondary billboard task do suggest a slight increase in workload or possible depletion of cognitive resources, such as that commonly found in vigilance tasks. However, that result was marginally significant.

Alpha-band showed a more complex pattern than RT over the session, with an overall increase in power over the driving session, interrupted by a temporary drop in power in the 4th time period. Other investigations of alpha-band activity during vehicle operation have found increases in alpha-band power over time. Simon et al. (2011) observed an increase in alpha-band over a driving session between the first 20 min of driving and the last 20 min. That was measured only in people who claimed to be very fatigued. Craig et al. (2012) found increases in alpha-band power at frontal, central, and posterior regions over time as participants engaged in a monotonous simulated driving task. A literature review by Lal and Craig (2001) concluded that alpha band activity changed as drivers become fatigued. Since we had participants engage in a fully autonomous drive, it is possible that some became passively fatigued or drowsy during the session. An attempt by the participant to maintain engagement despite the passive nature of monitoring the automation may partially explain the high accuracy of detection of the Unreliable Automation Arrows. Further, attention to a spatial location (Worden et al., 2000) and to features (Snyder and Foxe, 2010) also modulates alpha-band activity when participants are required to detect changes in the spatial location or visual features of stimuli when they are actively suppressing irrelevant stimuli. This has been observed over dorsal areas when the color was cued but over ventral areas when motion was cued (Snyder and Foxe, 2010). In the present study in which participants were required to discriminate stimuli defined by color, the modulation of alpha-band activity could, therefore, reflect the anticipated need to discriminate based on color.

We speculate that the interaction between alpha-band, HRV, and time period that was observed in the LME model may reflect changed influences of workload and/or attention over time. The increase in alpha-band activity from time period 4 to time period 5 may reflect lapses of attention to the arrow task during the last time period. This is similar to previous findings from O’Connell et al. (2009) in which they report increased alpha-band activity prior to missing a target. As discussed above, the increase in HRV may reflect the response to workload demands placed on participants. This increase in workload in addition to reduced attention may have affected participants’ response times to the Unreliable Automation Arrows indicating that the automation was in an unreliable state.

In contrast to previous work, we did not find that eye gaze measures predicted RT to signals of an unreliable automation state. Greater concentration of gaze (lower variance) has been associated with a higher workload (Victor et al., 2005). He et al. (2011) found that smaller horizontal gaze dispersion was an indication of mind wandering. As horizontal gaze dispersion did not contribute to modeling RT in the present study, we speculate that the billboard task and DEQs forced participants to maintain awareness of stimuli in the road environment and thereby remain attentive to the driving task. Further, the problem of “looking but not seeing” in driving may limit the usefulness of gaze concentration as a monitor of driver attentional state in the real world. In a real-world driving environment, operators may be less likely to detect a signal if they are not familiar with the automation. Further, in the current study, the reliability cue was not continuous. Rather, it appeared and remained on for a discrete amount of time (150 ms). This familiarity and the sudden onset of the cue likely heightened participants’ awareness of the Unreliable Automation Arrows and could have contributed to the high discrimination accuracy since participants were expecting the arrows to appear. In future studies, it would be useful to examine detection performance when changes were more gradual in a continuous display.

The present study has several limitations. First, driving in a simulator differs in a number of ways from on-road driving and the present design was an automated lane-changing task which did not require any active driving. Therefore, during the simulated drive, participants did not need to respond to sudden events common in everyday driving such as behavior of other drivers or pedestrians. Participants only needed to complete the tasks given to them. Further, the arrow task required participants to frequently monitor the automation display which changed the role of the driver from being an active participant to being a monitor of the automation. Monitoring the automation display, in conjunction with the secondary tasks, may have introduced additional noise making horizontal gaze dispersion less sensitive to operator state. We would note, however, that current SAE 2 vehicles do require the driver to monitor the automation display frequently. A second limitation was the absence of a measure of workload which makes it difficult to interpret the slowing of RT over time periods of the simulated drive. Third, the interruption of driving every 10 min makes the present study more relevant to city driving than to highway driving. Fourth, this study used a low-fidelity desk-top driving simulator. In future work, a high fidelity motion-based simulator with better automation capabilities allowing for longer automated drives will be used. Fifth, it could be argued that the high accuracy of target discrimination is a limitation. However, making the icons harder to discriminate would not be consistent with real-world driving demands which requires signals from an automation interface to be easily discriminable. Moreover, the speed of responding to those signals is an appropriate measure for driving performance. Despite these limitations, the present study provides insight into the feasibility of using portable, low-cost physiological measures to assess driver state in operational environments, including automated driving.

In sum, both EEG alpha-band and the interaction of HRV with alpha-band successfully modeled drivers’ readiness to respond to signals of automation unreliability. This suggests that both those measures reflect the ability to attend to important events during driving. Our results suggest that cardiac metrics obtained from low-cost wearable sensors can be further developed for in-vehicle monitoring of driver state. Such monitoring could be used to tailor alerts or even turn off the automation (as in certain General Motors models) if the operator is judged to not be attending sufficiently to the road or monitoring the automation.

## Ethics Statement

This study was carried out in accordance with the recommendations of The GMU Internal Review Board with written informed consent from all subjects. All subjects gave written informed consent in accordance with the Declaration of Helsinki. The protocol was approved by the The GMU Internal Review Board. No vulnerable populations were included.

## Author Contributions

DC was involved with the study design, data collection, data analysis, and write-up of this study. CB and PG were involved with the design, analysis, and write-up of this study. RM and DR aided in the study design.

## Conflict of Interest Statement

RM was employed by company Northrop Grumman. The reviewer BS and handling Editor declared their shared affiliation. The remaining authors declare that the research was conducted in the absence of any commercial or financial relationships that could be construed as a potential conflict of interest.

## Footnotes

^1^https://github.com/sccn/labstreaminglayer
